# Relationship between Body Composition and Serum Immunoglobulin Concentrations after Administration of Intravenous Immune Globulin–Preclinical and Clinical Evidence

**DOI:** 10.3390/pharmaceutics15020510

**Published:** 2023-02-03

**Authors:** Luigi Brunetti, Helene Chapy, Ronald G. Nahass, Rebecca Moore, Andrew Wassef, Derek Adler, Edward Yurkow, Leonid Kagan

**Affiliations:** 1 Department of Pharmacy Practice, Ernest Mario School of Pharmacy, Rutgers, The State University of New Jersey, Piscataway, NJ 08854, USA; 2 Department of Pharmaceutics, Ernest Mario School of Pharmacy, Rutgers, The State University of New Jersey, Piscataway, NJ 08854, USA; 3 Center of Excellence in Pharmaceutical Translational Research and Education, Ernest Mario School of Pharmacy, Rutgers, The State University of New Jersey, Piscataway, NJ 08854, USA; 4 IDCare, Hillsborough, NJ 08844, USA; 5 Molecular Imaging Center, Rutgers, The State University of New Jersey, Piscataway, NJ 08854, USA

**Keywords:** IVIG, primary immunodeficiency, immune globulin, body composition

## Abstract

The purpose of this study was to investigate the effect of obesity on immunoglobulin G (IgG) pharmacokinetics in a rat model of obesity, and to collect clinical evidence for an association between the body composition and intravenous immune globulin (IVIG) pharmacokinetic parameters in humans. In a preclinical study, pharmacokinetics of human IgG was evaluated after intravenous (IV) and subcutaneous (SC) delivery to obese and lean rats (n = 6 in each group). Serial serum samples were analyzed using an ELISA. The animal body composition was assessed using computer tomography. Patients with primary immunodeficiency currently managed with IVIG, and at a steady state, were enrolled in the clinical study (n = 8). Serum immune globulin (Ig) concentrations were measured at baseline and immediately after the administration of two consecutive treatments, with an additional measurement at two weeks after the first administration. In addition to the patient demographic and clinical characteristics, body composition was measured using bioelectrical impedance analysis. The pharmacokinetics of human IgG was significantly different between the obese and lean rats after both the IV and SC administration of 0.5 g/kg. Furthermore, a significant difference in endogenous rat IgG was observed between the two strains. In the human study, total serum IgG and subtype (IgG1, IgG2, IgG3, IgG4) half-life negatively correlated with the body mass index and fat mass. The mean change in the total serum IgG concentration was significantly correlated to body mass index and fat mass. The results of the studies corroborated one another. In the animal study, most pharmacokinetic parameters of human IgG following IV and SC administration were significantly affected by obesity and changes in the body composition. In the clinical study, the mean serum IgG change after the IVIG administration strongly correlated to the BMI and body fat mass. Future studies are needed to establish the outcomes achieved with more frequent dosing in obese individuals with primary immunodeficiency.

## 1. Introduction

Approximately 250,000 patients in the United States are diagnosed with primary immunodeficiency, and immune globulin G (IgG) replacement is the mainstay of therapy [1]. Current dosing practices for intravenous immune globulin (IVIG) may be inadequate for extreme body weight [2,3,4]. Actual, ideal, and adjusted (taking a portion of the excess body weight above ideal body weight, usually 40%, and adding to the ideal body weight) body weight-based dosing strategies are suggested in the literature [4,5,6]. These recommendations are based on expert opinion rather than high-quality evidence. Adopting a specific strategy for dosing is highly variable, depending on the clinician and institutional setting [5]. In the U.S., many payors have adopted strategies to reduce IgG therapy costs by capping doses. These recommendations are often based on the presumption that IgG distribution is limited to the vascular space [7]. While this assertion is logical, it does not account for the changes adipose tissue may confer on target sites nor for the adipose tissue’s potential to function as a metabolic sink or a source of inflammatory mediators [8]. The latter would be especially important in patients receiving SC immune globulin administration.

Several observational studies have evaluated IgG dosing in obese patients [2,9,10,11], and are often cited to support dosing strategies. Many of these studies were not representative of the general population, contained a wide variety of patients with different IgG indications, and had sparse serum sampling. Moreover, IgG distribution is not only a function of the partition coefficient, but may be influenced by active transport and inflammation [8].

Using actual body weight for the dose calculations is the current recommendation in the FDA-approved prescribing information for IVIG products. Using actual body weight to dose IgG in obese patients may increase the risk of thrombosis, due to the increased blood viscosity, activation of platelets, or vasospasm [9,10]. Increased blood viscosity has been reported as IgG dose-dependent [9,12,13]. The use of ideal or adjusted body weights have been advocated to reduce the side effects and drug expenditures [2]. The impact of using measures of body weight other than the actual body weight to calculate IgG doses on clinical outcomes and the effect of obesity on IgG pharmacokinetics has not been experimentally evaluated. Although some data suggest actual body weight for IgG dosing, in a survey (2015) of 92 academic institutions in the United States, approximately 60% of respondents indicated they do not use the actual body weight to dose IgG [5]. This choice is likely related to the cost of IgG treatment, which tremendously impacts the healthcare system. The cost of IVIG therapy for patients with primary immunodeficiency is approximately $30,000 per patient annually [14], which corresponds to a total of more than $3.5 billion/per year for primary immunodeficiency alone (there are more than 150 unlabeled uses for IgG reported in the literature) [15]. Using the ideal or adjusted body weight for dosing may conserve IgG [16]. To this end, the doses are frequently rounded to the nearest vial size [17].

The mechanisms by which obesity affects the pharmacokinetics of protein therapeutics, including IgG, have not been sufficiently investigated. It was recently suggested that IgG should be dosed based on the patient response in primary immunodeficiency rather than the body weight [3]. However, even this approach involves a loading dose based on ideal body weight, and then an adjustment of 0.15 mg/kg/month when the patient presents with a severe infection or three or more moderate infections over one year [3]. This assertion has been challenged by some authors who suggest that the adjusted body weight is more appropriate for individuals with obesity [4]. Furthermore, dosing recommendations (initiation and adjustment) were not based on robust clinical data but rather on the presumption that the volume of the distribution of IgG is limited to the intravascular space. This study’s goals were to investigate the effect of obesity on IgG pharmacokinetics in a rat model of obesity and to collect clinical evidence for an association between body composition and IVIG pharmacokinetic parameters in humans.

## 2. Materials and Methods

### 2.1. Preclinical Study

#### 2.1.1. Materials

Human IgG (10% Liquid, Privigen^®^) was obtained from the Cancer Institute of New Jersey (CINJ, New Brunswick, NJ, USA). The DietGel 76A and HydroGel were obtained from ClearH_2_O Inc. (Portland, ME, USA). Meloxicam, carprofen, and bupivacaine were obtained from Rutgers Animal Facility. The jugular vein catheters were made from PE50 tubing purchased from Braintree Scientific Inc (Braintree, MA, USA).

#### 2.1.2. Animals

The study was conducted in accordance with an approved protocol from the Institutional Animal Use and Care Committee of Rutgers, the State University of New Jersey. The Male Obese Prone (Crl:OP(CD)) and Obese Resistant (Crl:OR(CD)) were purchased from Charles River (Wilmington, MA, USA). Both strains were developed from outbred Crl:CD(SD) rats, and obese prone (OP) rats were shown to become obese when fed a high-fat diet, despite having a fully functional leptin receptor, while the obese resistant (OR) rats did not become obese. The rats were received at six weeks of age, weighing around 190 g (OP) and 150 g (OR). Upon arrival and during the study, the animals were housed in pairs with free access to water and maintained on a 12/12 h dark/light cycle. To maximize the weight difference between the strains, OP rats were fed a high-fat diet (D12492, 60 kcal%), and OR rats were fed a control diet (D12450J, 10 kcal%) from Research Diet Inc (New Brunswick, NJ, USA).

#### 2.1.3. Assessment of Body Size and Composition in Animals

The total body weight (TBW) of each rat was recorded every week from arrival to the end of the study. At the time of the dosing of the human IgG (20 weeks of age), the OP rats weighed around 520 g and the OR rats around 350 g.

The body composition of animals (% fat mass) was measured by computer tomography (CT) at the Rutgers Molecular Imaging Center using the Albira PET/CT system from Bruker (Billerica, MA). All rats were fasted the night before the CT imaging to prevent food artifacts in the gut and possible issues with general anesthesia. Isoflurane was used as the general anesthetic (4–5% for induction and 1–2% for maintenance during the scan). After being anesthetized and weighted, the rat was placed prone in the CT cradle with the rat’s head secured into the isoflurane nose cone. Care was taken to ensure all parts of the rat were contained in the cradle, with excess skin tucked and secured before scanning. The scan time was approximately 45 to 60 min, utilizing four to five beds depending on the rat size. The imaging protocol used for the CT scan was called the “Good setting” with 400 slices per bed with a Low Dose (200 mA) and High Voltage (45 kV). When the scans were completed, the data were converted into the “Best setting”, which provided a sharper image with better contrast using 600 slices. The results were then transferred to the InVicro Vivoquant software (InVicro, Boston MA) for analysis. Using the different signal shades of gray (or pixel intensity, from −1000 to 1600), the intensities of different body tissues were recorded: Miscellaneous (−800 to −500), Fat (−500 to −200), Muscle (−200 to 125), and Bone (125 to 1600). The total counts were completed for each intensity segment, and mm^3^ was converted into grams. Bone and fat were then adjusted by density, 1.9 and 0.9, respectively; the other tissues (muscle and miscellaneous) did not need adjusting (density of 1) [18]. The total body weight was determined by adding up the total mm^3^/grams from each segment and then compared to the scale body weight taken before scanning; on average, the digitally derived weights were +/− 10% of the actual body weights. From this data, the percent fat to body weight was calculated. The body composition was measured at 7, 13, and 20 weeks of age.

#### 2.1.4. Experimental Procedure

The OP and OR rats were randomly divided into groups (n = 6 each) depending on the mode of administration, intravenous (IV) or subcutaneous (SC). In the IV groups, animals were instrumented with a right jugular vein PE50 catheter to allow for the IV infusion administration. The surgery was performed under isoflurane anesthesia, and SC meloxicam or carprofen and intradermal bupivacaine were provided for analgesia. After surgery, the animals were allowed to recover for 24–48 h. 

The IV dosing of the human IgG (0.5 mg/kg, 10% Liquid, Privigen^®^) was performed in awake rats using a Harvard Apparatus 70–3005 PhD Ultra infusion pump at a 1 mL/h flow rate and lasted about 2–3 h. The SC administration of human IgG (0.5 mg/kg) was performed in lightly anesthetized rats (isoflurane) at the back of the animals. The blood samples (50 μL) were obtained after the end of infusion or after the SC administration (up to 6 weeks post-dose) from the saphenous vein in the anesthetized rats (isoflurane). The samples were taken at 1, 5, 8, 24, and 72 h, and then every week up to 6 weeks after the IV dosing, and at 1, 5, 8, 12, 48, 72, and 96 h, and then every week up to 6 weeks after the SC dosing. The blood samples were allowed to clot for 30–60 min at room temperature and were centrifuged (6000 rpm for 7 min), aliquoted, and stored at −80 °C until analysis of the human IgG concentrations. The samples were also collected from animals in IV groups pre-dose (at 7, 14, 17, and 20 weeks of age) and post-dose (21, 22,23, and 26 weeks of age), and analyzed for endogenous rat IgG.

### 2.2. Clinical Study 

#### 2.2.1. Population

The male and female patients aged 18 to 75 years receiving the institutional standard IVIG dosing (400–600 mg/kg) were eligible for the study enrollment. All patients who received IVIG for at least six months were considered to be at a steady state. The serum IgG concentration at the steady state after IV administration is typically achieved after the fourth to sixth infusion. Demographic and clinical data were obtained at the time of enrollment [19]. Patients with liver impairment (elevations in liver enzymes of greater than three times the upper limit of normal) or reduced renal function (CrCl < 50 mL/min) were excluded. Patients with a pacemaker or an automatic implantable cardioverter-defibrillator were not eligible to participate. These exclusions were necessary because the bioelectrical impedance analysis (BIA) device may interfere with these medical devices. The Rutgers Biomedical and Health Sciences Institutional Review Board approved (PRO20160000739) the study. All study subjects consented prior to participation.

#### 2.2.2. Assessment of Body Size and Composition in Humans

Actual body weight and height were measured at the time of enrollment. Body mass index, body surface area, ideal, lean (Janmahasatian equation) [20], and adjusted body weight were calculated using standard equations [21]. The body composition of all subjects was estimated using a multi-frequency segmental body composition analyzer (BIA, Tanita MC-780U), per the manufacturer’s instructions. This method estimates the body fat percentage, body fat mass, BMI, fat free mass, estimated muscle mass, total body water, and basal metabolic rate by measuring the body resistance to the current. These measurements were used to estimate the body composition using the built-in proprietary equations. The BIA is the most studied bedside technique for assessing body composition, as it is affordable, and easy to transport and use [22,23]. Although similar to other body composition measurements, it does not directly measure the body composition, it measures the resistance of the body tissues to an electric current, an indirect measure of the body composition [22,23]. The level of precision produced by BIA is reported as good, with a 1-to-2% variability between the repeat measures [24,25]. Thompson et al. found good absolute and relative agreement between the changes in the body composition assessed by the dual-energy X-ray absorptiometry (DXA) and BIA, with small biases in the estimation of the fat mass and percentage body fat in overweight women [26].

#### 2.2.3. Study Design

Serum immunoglobulin measurements were taken immediately before and after the IVIG administration on two consecutive treatments (trough 1, peak 1, trough 2, peak 2; approximately one month apart). Another measurement was taken at approximately two weeks of post-infusion (after treatment 1). Briefly, 6 mL of blood was collected in a serum vacutainer, blood was allowed to clot, and the serum was separated by centrifugation at 3000 g for 10 min. The samples were divided into aliquots and stored at −80 °C until analysis. 

### 2.3. Bioanalytical Procedure

Human IgG and rat IgG in the rat serum were quantified using the human IgG SimpleStep^®^ ELISA kit (ab195215; Abcam, Cambridge, MA, USA) and the rat IgG SimpleStep^®^ ELISA kit (ab189578; Abcam, Cambridge, MA, USA), respectively. The assays were performed, as indicated in the kit protocols. The calibration curves were fitted using GraphPad Prism 6 and a four-parameter logistic equation. The precision and accuracy were within ±20%. The working ranges of the human IgG and the rat IgG assay were 0.00023–150 mg/mL and 0.3125–20 mg/mL, respectively. 

For serum samples in the human study, the IgG subtypes, IgA, IgE, and IgM, were measured using a magnetic bead multiplex assay (Antibody Isotyping 7-Plex Human ProcartaPlex™ Panel, Invitrogen, Carlsbad, CA, USA) on a Luminex platform (Magpix, Thermofisher, Waltham, MA, USA) according to the manufacturer protocol. The total serum IgG concentration was calculated as a sum of the four IgG subtypes.

### 2.4. Data Analysis

In the preclinical study, the rat body weight and composition measurements and rat and human IgG serum concentration data were presented as the mean ± standard deviation (SD). Standard noncompartmental analysis was performed for the human IgG concentration-time data using Phoenix WinNonlin 8.3 (Certara, Princeton, NJ, USA). The bioavailability following SC administration was calculated by dividing the individual AUC values by the mean AUC following IV administration. Student’s *t*-test (*p* < 0.05) was used to compare the data between the lean and obese groups statistically.

For the clinical study, all data were analyzed using descriptive statistics. The counts and proportions were calculated for all the categorical data, and the mean and standard deviation for the continuous data. The mean change in the total serum IgG and subtypes of IgG was calculated by subtracting the trough serum concentration from the peak concentration on each occasion for each subject and calculating the mean. The mean trough and peak total serum IgG and subtypes were obtained by calculating the mean of each of the available troughs and peak serum IgG. Pearson’s correlation coefficient was calculated to describe the correlation between various measures of body composition and IgG pharmacokinetic parameters. The total serum IgG and subtype half-life was calculated for each subject using Phoenix Winnonlin (v8.3, Certara, Princeton, NJ, USA) using peak 1, 2-week measurement, and trough 2. The change in the serum Ig concentration after each administration was evaluated using the paired *t*-test

## 3. Results

### 3.1. Preclinical Study

**Figure 1:**
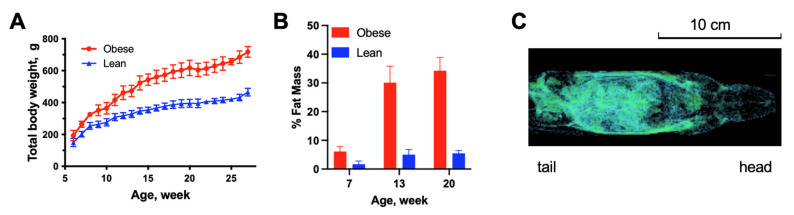
Difference in body weight and composition between obese (OP) and lean (OR) rats. Panel (**A**)—total body weight. Panel (**B**)—percentage of fat mass in body weight as determined by CT scan. Panel (**C**)—visualization of fat disposition (blue-green) in a rat body in a representative CT image. Data are shown as mean ± SD (n = 12 each group). Total body weight and percentage of fat mass were significantly different between obese and lean animals at all time points (*p* < 0.05, *t*-test).

In this study, the effects of obesity on pharmacokinetics on the human IgG and endogenous rat IgG were evaluated in the obese-prone (OP) and obese-resistant (OR) rat strains derived from the same ancestral strain. The animal growth curves are shown in Figure 1A. The total body weight was significantly different between the groups (*p* < 0.05) from the beginning of observation at the age of 6 weeks. At the time of the dosing of the human IgG (20 weeks of age), the OP rats weighed around 520 g and OR rats around 350 g. The body composition of animals was evaluated using CT imaging at three time points (7, 13, and 20 weeks of age), as shown in Figure 1B. The percentage of body fat was significantly different between groups (*p* < 0.05) at all time points; and, at the time of dosing of human IgG, the mean fat percentage was 34% in OP and 5.5% in OR rats. Figure 1C shows a representative CT image of the fat distribution in the rat body.

**Figure 2:**
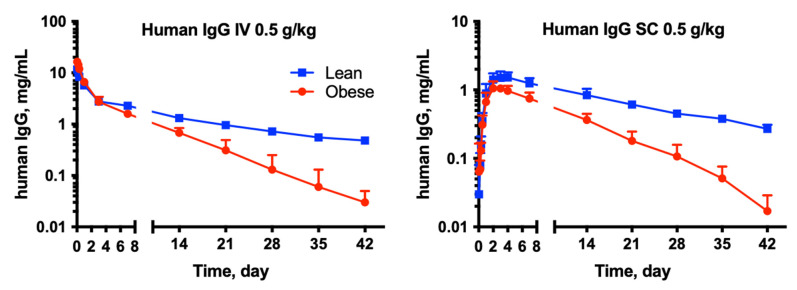
Time-course of human IgG following IV and SC administration of 0.5g/kg dose to obese (OP) and lean (OR) rats. Data are shown as mean ± SD (n = 6 each group).

The pharmacokinetics of human IgG was significantly different between the OP and OR rats after both the IV and SC administration of 0.5 g/kg. The mean serum concentration-time profiles are shown in Figure 2 and pharmacokinetic parameters are presented in Table 1 and Table 2. Following IV dosing, the obese rats showed significantly lower AUC, 3-fold shorter terminal half-lives, lower volume of distribution (in mL/kg) and higher clearance (in mL/day/kg) compared to lean rats. However, C_max_ (as measured immediately after IV infusion) was significantly higher in the obese group. Following SC administration, the obese rats showed significantly lower AUC and shorter terminal half-life than the lean rats. C_max_ was also lower in the obese rats; however, T_max_ was comparable. Furthermore, the SC bioavailability was lower in obese rats (32 vs. 54%).

**Figure 3:**
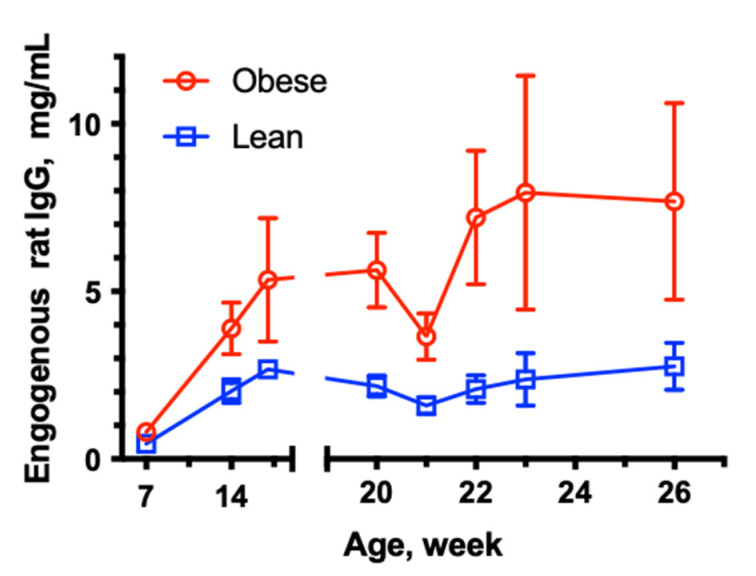
Time-course of endogenous rat IgG in obese (OP) and lean (OR) rats. Data are shown as mean ± SD (n = 6). There was a statistical difference between groups at each time point (*p* < 0.05, *t*-test).

The concentration of endogenous rat IgG was compared between the OP and OR animals (only animals in IV groups were tested, n = 6) as shown in Figure 3. The endogenous rat IgG was significantly higher in obese rats at all tested time points. Within the group, the variability was higher in obese animals. At the time of the human IgG dosing, the concentration of rat IgG was 2.5-fold higher in obese animals. Interestingly, the concentration of rat IgG temporarily decreased (more pronounced in obese group) after the administration of 0.5 g/kg of human IgG. This finding could be related to the competition between rat and human IgG to the binding to neonatal Fc receptor (FcRn) and the saturation of FcRn-mediated recycling of IgG by high exogenous dose of human IgG.

### 3.2. Clinical Study

**Figure 4:**
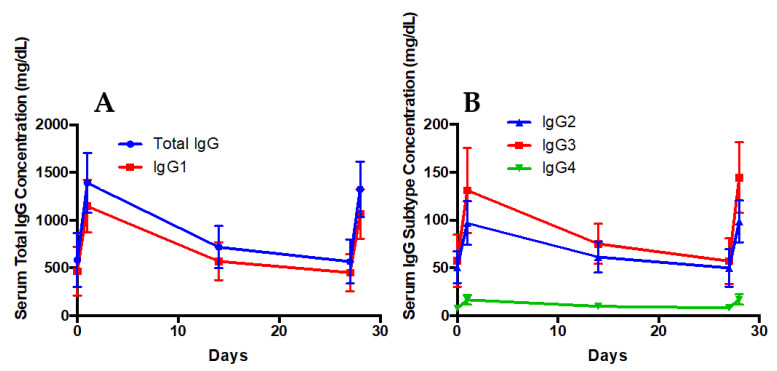
Serum total IgG and subtype IgG1 (Panel (**A**)) and subtypes 2–4 (Panel (**B**)) profiles after dosage administration on days 1 and 28. Error bars represent standard deviation. Changes in all serum immunoglobulin concentrations after administration of IVIG were significant (*p* < 0.05).

**Figure 5:**
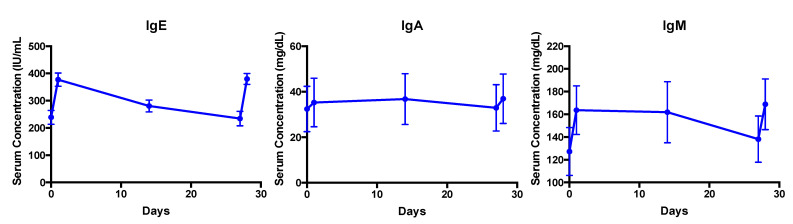
Serum IgE, IgA, and IgM concentration after intravenous immune globulin (IVIG) dosage on days 1 and 28. Changes in all serum Ig concentrations after IVIG administration were significant (*p* < 0.05) with the exception of IgA at the first IVIG administration.

A total of eight subjects with primary immunodeficiency (common variable immune deficiency, CVID) were included in this pilot study, and the mean weight normalized IVIG dose administered was 0.413 g/kg (dose/actual body weight) monthly. Table 3 provides a summary of the patient characteristics. The mean age was 63 years, and 6 of 8 subjects were female. The median BMI was 26.8 kg/m^2^ (range 20.5–34.4 kg/m^2^). Body composition was estimated using BIA on two separate occasions (on day 1 and approximately 30 days later) and displayed low variability between the measurements. Table 4 summarizes the mean serum trough, peak, and change after the IgG administration in the total IgG and each subtype. As expected, IgG_1_ was the most abundant subtype. The mean half-life was the longest in the IgG_2_ and IgG_4_ subtypes. Various descriptors of the body composition were correlated with the IgG pharmacokinetic parameters, and those that were significant are summarized in Table 5. The total serum IgG half-life displayed a negative correlation with the body mass index and fat mass, but did not reach significance. Other body composition descriptors were tested; however, no significant correlations were identified. The mean change in the total serum IgG concentration was significantly correlated to the body mass index and fat mass. The trend in IgG and subtype response after IV IgG administration is illustrated in Figure 4. Serum IgA, IgE, and IgM concentrations were also evaluated, as shown in Figure 5. A small change in IgA and IgM was observed, with a more significant change in serum IgE concentration after administering the commercially available IVIG. With the exception of IgA at the first IVIG infusion evaluated, a significant change in the serum IgA (mean change first infusion 2.8 ± 6.6 mg/dL, *p* > 0.05; second infusion 4.0 ± 3.4 mg/dL, *p* < 0.05), IgE (mean change first infusion 138.4 ± 59.4 mg/dL, *p* < 0.05; second infusion 145.4 ± 67.5 mg/dL, *p* < 0.05), and IgM (mean change first infusion 36.3 ± 39.4 mg/dL, *p* < 0.05; second infusion 30.8 ± 24.4 mg/dL, *p* < 0.05) concentrations were observed versus the baseline after each administration of IVIG.

## 4. Discussion

There is an urgent clinical need to develop approaches for the optimization of IgG dosing in the obese population. Approximately 25% of individuals with primary immunodeficiency are obese [27]. Understanding the effects of obesity on IVIG exposure and treatment outcomes is critical. Data examining the impact of obesity on primary immunodeficiency outcomes are scarce, and very few studies have evaluated drug concentrations prospectively. One study reported an increase in sepsis (12% versus 6%; *p* = 0.05) in patients with obesity and common variable immune deficiency versus normal-weight patients [28]. Other studies suggest no difference in IgG exposure between obese and non-obese individuals [8].

There are various potential concerns with IgG disposition in obesity. First, IgG is a polar molecule with a small distribution volume. As such, IgG would not distribute into the adipose tissue but rather accumulate preferentially in the plasma [29]. IgG distribution is complex and can be influenced by both inflammation and active transport [30]. Notably, obesity is associated with chronic inflammation. FcRn protects IgG from lysosomal degradation through a recycling process in which IgG is circulated into an intracellular protein reservoir and eventually back into circulation [31]. The expression of FcRn is lower in adipose tissues versus other tissues [32]. Catabolism of IgG may also be increased due to a large number of activated macrophages in the excess adipose tissue. The primary elimination route for IgG is via the reticuloendothelial system [33]. As such, the half-life of IgG may be reduced in obese individuals relative to lean individuals.

In this study, we performed both a preclinical assessment and a pilot clinical study to evaluate the effect of obesity and changes in body composition on the pharmacokinetics of IgG. The results of both studies corroborated each other and demonstrated the effects of obesity on the IgG disposition. In the preclinical study, almost all pharmacokinetic parameters of human IgG were significantly different in the obese rats compared to the lean rats following IV and SC administration (when animals were dosed on a mg/kg basis), as shown in Table 1 and Table 2, and Figure 2 and Figure 3. Overall, obesity resulted in lower exposure (AUC), faster clearance, lower volume of distribution, a much shorter half-life, and significantly reduced bioavailability following SC dosing. Furthermore, endogenous rat IgG concentrations were higher in obese rats, possibly related to an obesity-induced inflammatory state.

In the pilot clinical study, the IgG pharmacokinetic parameters correlated with body composition after the IV administration. The half-life for IgG decreased as BMI and body fat mass increased. These results are supported by findings in our preclinical study (3-fold shorter half-life in the obese group). These findings suggest that the optimal dosing interval for individuals with obesity may be shorter than for non-obese individuals. While clinical findings did not reach statistical significance (likely due to the small size of populations and limited range of BMI), they warrant attention in larger prospective studies. There was variation in the half-life of each IgG subtype. A previous radioactive tracer study in individuals with near normal serum IgG reported IgG_3_ to have the shortest half-life at 7 days [34]. The calculated half-life for IgG_3_ in our study was approximately 20 days. While a significant variation from the report above, several other studies measuring IgG subtype serum concentration in individuals with immunodeficiency report IgG_3_ half-life in the range of 15.7 to 49.9 days [35,36,37,38]. This observation requires further investigation. Notably, the mean change in the serum IgG concentration was significantly correlated with the BMI and body fat mass. These findings suggest that individuals with obesity have a greater increase in the serum IgG concentration following the administration of standard doses. Our preclinical data corroborates these results, as the initial concentrations after the human IgG IV infusion in rats were higher in the obese group. Hyperviscosity after the administration of IVIG may be more pronounced in individuals with a greater change in serum IgG concentration from the baseline [39,40]. Additional data are needed to confirm this hypothesis. Collectively, these data suggest that lower and more frequent dosing may be ideal in this patient population, a postulation that requires evaluation in further larger studies. 

IVIG infusions are typically administered every 3 to 4 weeks at an initial dose of 300 to 800 mg/kg. All patients in this study were administered IVIG every four weeks, and the mean mg/kg dosage was within this range. None of the patients included in the study experienced treatment failure or adverse events. The current recommendations suggest an IgG trough concentration of at least 500 mg/dL, and some clinicians prefer to target 300 mg/dL above the patient’s pretreatment concentration [41]. All of the patients in this study were above the 500 mg/dL threshold. In agammaglobulinemia, a form of primary immunodeficiency, IgG trough concentrations above 800 mg/dL prevented severe bacterial infections [42]. While IgG concentration is a surrogate for success, the treatment adjustments should not be based solely on this value, but preferably on the patient’s symptoms or response to therapy [3].

We also measured serum IgA, IgM, and IgE at the specified time points throughout the study. There was a small but significant change in IgA or IgM after the IVIG administration; however, there was a substantial change in IgE. Our finding related to IgE were unexpected and warranted further investigation. The IgE-mediated anaphylaxis characterized by flushing, facial swelling, dyspnea, hypotension, loss of consciousness, nausea, and vomiting has been reported in patients treated with IVIG, in individuals with IgA deficiency, or those with anti-IgA antibodies of the IgE type [43]. These reactions typically occur within minutes to several hours after the IVIG infusion. None of the patients in our study experienced these symptoms. Less severe dermatological reactions such as rash or hives can occur and have been attributed to components or an allergen present in the IVIG preparation not removed during the manufacturing process. What is unknown is whether the administration of IVIG (including IgE) confers a Prausnitz-Kustner-like reaction or passive blood anaphylaxis contributing to the dermatological reactions, as has been suggested with SC immunoglobulins [44]. 

None of the commercially available IVIG products list IgE content on their FDA-approved product labeling. The primary immune deficiencies are associated with elevated IgE concentrations, including Job syndrome, immune dysregulation, polyendocrinopathy, enteropathy, X-linked (IPEX), Wiskott-Aldrich syndrome, Omenn syndrome, and atypical complete DiGeorge syndrome [45]. Elevations in IgE are possible in this population; however, we observed the elevations immediately after the IVIG administration. Only a few reports measured the IgE in commercially available IVIG products and the change in serum IgE concentration after the IVIG administration [46,47]. Paganelli and colleagues observed a wide range of IgE in various products and within different lot numbers of the same product (ranging from approximately 20 to 900 IU/mL per 5 to 6 g of IVIG product). Similarly, Toro and colleagues reported varying amounts of IgE in commercially available products after observing a change in the serum IgE after IVIG in children with hypogammaglobulinemia [46]. IVIG has been postulated to decrease the IgE production in vitro [48], but this finding has yet to translate to human studies [49]. Further longitudinal studies are necessary to confirm whether the repeated administration of IVIG results in short-term elevations in IgE, and whether the elevations are associated with adverse reactions. While IgG represents more than 90% of the proteins present in IVIG, trace amounts of IgA, IgE, IgM, and other immune globulins may also be present [50]. 

Some important limitations of the clinical pilot study should be acknowledged. First, the study’s small sample size limits robust analyses, and the results require confirmation in larger cohorts. While we provide some clinical evidence supporting a reduced half-life of IgG with increasing body fat mass and body mass index, this finding did not reach statistical significance. However, preclinical data strongly supports the clinical results. BIA is imperfect and prone to errors in estimating the body composition. While BIA was completed on two separate occasions and the results demonstrate little variability between visits, the external variables may influence the accuracy of the body composition measurement. The hydration of fat free mass has been proposed to be approximately 0.73 (range 0.69–0.77) [51], and two of the subjects enrolled had hydration of fat free mass that deviated significantly from this range. Both of these individuals had cardiometabolic disease. Factors that impair the body fluid balance (i.e., edema and diuretic therapy) are commonly seen in cardiometabolic disease and may influence the estimation of the body composition. Importantly, edema may cause a false high fat free mass [52]. The BIA derived fat free mass was higher than the Janmahasatian method for estimating fat free mass in the majority of cases. As a result, the associations between fat free mass and IVIG pharmacokinetics should be interpreted cautiously. Moreover, IVIG had been previously titrated to achieve the target steady-state IgG concentration (serum IgG concentration > 500 mg/dL) in the study population. Nonetheless, this study’s finding may apply to individuals starting IVIG since this population is at greater risk of dosing inadequacy and adverse reactions. 

## 5. Conclusions

The effects of obesity on the pharmacokinetics of IgG were investigated in the preclinical setting and a pilot clinical study, and the results were corroborated. In the animal study, most pharmacokinetic parameters of human IgG following IV and SC administration were significantly affected by obesity and changes in the body composition. In the clinical study, the mean serum IgG change after IVIG administration strongly correlated to BMI and body fat mass. These studies suggest that the effects of obesity on IgG pharmacokinetics might require dose level and frequency of administration adjustment in the obese population, and these approaches should be further evaluated in future studies. The results of this pilot study highlight the importance of individualized therapy and suggest that larger prospective human studies are needed to assess the clinical impact of altered IgG pharmacokinetics.

## Figures and Tables

**Table 1:** Pharmacokinetic parameters of human IgG following IV delivery of 0.5 g/kg to obese (OP) and lean (OP) rats.

Parameter	Lean		Obese	
	Mean	SD	Mean	SD
T_1/2_, day	18.8	2.1	6.0 ^#^	1.6
C_max_, mg/mL	10.1	4.1	16.5 ^$^	1.9
AUC, day·mg/mL	68.8	11.3	41.0 ^#^	6.5
V_ss_, mL/kg	173.9	45.9	77.6 ^#^	6.4
CL, mL/day/kg	7.5	1.6	12.4 ^#^	1.8

AUC—area under the serum concentration-time curve extrapolated to infinity; T_1/2_—terminal half-life; C_max_—maximal serum concentration (measured immediately after the end of infusion); V_ss_—volume of distribution at steady state; CL—systemic clearance. ^$^—statistically different from lean group (*p* < 0.01). ^#^—statistically different from lean group (*p* < 0.001).

**Table 2:** Pharmacokinetic parameters of human IgG following SC delivery of 0.5 g/kg to obese (OP) and lean (OP) rats.

Parameter	Lean		Obese	
	Mean	SD	Mean	SD
T_1/2_, day	19.5	2.9	7.1 ^#^	1.2
C_max_, mg/mL	1.6	0.3	1.1 ^$^	0.2
T_max_, day	3.2	0.8	2.8	0.7
AUC, day·mg/mL	37.5	3.0	13.2 ^#^	3.2
F (%)	54.4	4.3	32.3 ^#^	7.8

AUC—area under the serum concentration-time curve extrapolated to infinity; T_1/2_—terminal half-life; C_max_—maximal serum concentration; T_max_—time to reach C_max_; F—bioavailability. ^$^—statistically different from lean group (*p* < 0.01). ^#^—statistically different from lean group (*p* < 0.001).

**Table 3:** Individual and summary characteristics of patients with primary immune deficiency enrolled in the study.

Overall Characteristic	Overall	Individual Characteristics	Patient 1	Patient 2	Patient 3	Patient 4	Patient 5	Patient 6	Patient 7	Patient 8
Median age (range)	63 (58–71)	Age, years	71	57	55	67	61	72	58	63
Percent female	75	Sex	Female	Female	Female	Male	Male	Female	Female	Female
Mean dose, g/kg (SD)	0.41 (0.04)	Dose, g/kg	0.47	0.39	0.43	0.43	0.37	0.46	0.36	0.40
Product name	-	Product Name	Octagam	Gammagard	Octagam	Octagam	Octagam	Gamunex-C	Octagam	Octagam
Mean height, cm (SD)	161.6 (13.1)	Height, cm	144.8	165.1	163.8	175.3	182.9	148.6	152.4	160.0
Mean weight, kg (SD) Weight range, kg	71.9 (17.1) 43–93	Weight, kg	43	64	58	93	81	65	83	88
Ideal body weight **, kg (SD)	57.9 (9.4)	Ideal body weight, kg	46	60	59	68	74	49	51	57
Mean body mass index, kg/m^2^ (SD)	27.5 (5.8)	Body mass index, kg/m^2^	20.5	23.5	21.6	30.3	24.2	29.4	35.7	34.4
Mean body fat percentage (SD)	37.8 (8.7)	Body fat percentage	35.5	31.5	35.2	34.8	23.7	46.6	49.0	46.3
Mean fat mass, kg (SD)	27.3 (10.0)	Fat mass, kg	15.3	20.0	20.2	32.2	19.1	30.4	40.4	40.8
Mean fat free mass w/BIA, kg	44.3 (11.8)	Fat free mass, kg	31.80	40.90	37.90	65.10	63.10	37.80	43.60	47.50
Mean fat free mass w/Janmahastian 2005, kg	46.0 (12.1)	Fat free mass, kg	27.70	43.50	37.27	60.36	61.45	34.82	42.09	47.36
Total body water, kg (SD)	32.6 (7.8)	Total body water, kg	22.5	28.9	28.1	45.3	43.3	28.1	31.6	33.0
Basal metabolic rate, kCal (SD)	1346 (317.5)	Basal metabolic rate, kCal	882	1293	1138	1784	1770	1105	1331	1465
Indication for IVIG	-	Indication for IVIG	CVID	CVID	CVID	CVID	CVID	CVID	CVID	CVID

** Ideal body weight was calculated using the following equations: men, 50 + (0.91 × [height–152.4]); women, 45.5 + [height–152.4]). All other body metrics were obtaining using bioelectrical impedance analysis. BIA, bioelectrical impedance analysis; CVID, common variable immune deficiency; IVIG, intravenous immune globulin. No bronchiectasis or diarrhea reported in study subjects.

**Table 4:** Key pharmacokinetic parameters for total serum IgG and subtypes in humans.

Parameter	IgG_Total_	IgG_1_	IgG_2_	IgG_3_	IgG_4_
[C_min_], mg/dL ± SD	575.6 ± 254.7	460.0 ± 223.0	50.6 ± 18.5	58.0 ± 26.5	8.0 ± 3.3
[C_max_], mg/dL ± SD	1358.5 ± 295.3	1106.0 ± 261.2	97.8 ± 22.0	137.9 ± 39.5	16.9 ± 5.1
[Δ], mg/dL ± SD	783.0 ± 209.3	646.0 ± 180.8	47.2 ± 23.4	79.9 ± 23.6	8.9 ± 5.1
T_1/2_ ± SD	22.2 ± 7.4	21.2 ± 6.8	28.1 ± 8.8	21.0 ± 4.8	34.4 ± 20.8

C_min_ = trough serum concentration; C_max_ = peak serum concentration; t_1/2_ = half-life; Δ = change in serum concentration from trough to peak.

**Table 5:** Pearson’s correlation coefficient (95% confidence interval) between available IgG pharmacokinetic parameters and select descriptors of body composition in humans.

Body Mass Index					
Parameter	IgG_Total_	IgG_1_	IgG_2_	IgG_3_	IgG_4_
T_1/2_	−0.59 (−0.92, 0.19)	−0.55 (−0.25, 0.91)	−0.57 (−0.91, 0.22)	−0.77 * (−0.96, −0.15)	−0.50 (−0.89, 0.32)
[C_max_]	0.22 (−0.58, 0.80)	0.23 (−0.57, 0.80)	0.28 (−0.53, 0.82)	−0.07 (−0.71, 0.70)	0.22 (−0.57, 0.80)
[C_min_]	−0.36 (−0.46, 0.85)	−0.32 (−0.83, 0.50)	−0.37 (−0.45, 0.85)	−0.53 (−0.90, 0.27)	−0.29 (−0.52, 0.82)
[Δ]	0.75 * (0.09, 0.95)	0.72 * (0.03, 0.95)	0.55 (−0.25, 0.91)	0.49 (−0.33, 0.89)	0.41 (−0.42, 0.87)
Body fat mass					
T_1/2_	−0.54 (−0.90, 0.27)	−0.50 (−0.31, 0.89)	−0.48 (−0.88, 0.34)	−0.73 * (−0.95, −0.05)	−0.42 (−0.87, 0.40)
[C_max_]	0.29 (−0.52, 0.83)	0.30 (−0.51, 0.83)	0.34 (−0.48, 0.84)	−0.05 (−0.68, 0.73)	0.29 (−0.52, 0.83)
[C_min_]	−0.28 (−0.53, 0.82)	−0.23 (−0.57, 0.80)	−0.28 (−0.53, 0.82)	−0.53 (−0.28, 0.90)	−0.18 (−0.79, 0.60)
[Δ]	0.74 * (0.08, 0.95)	0.72 * (0.02, 0.94)	0.53 (−0.71, 0.71)	0.52 (−0.29, 0.90)	0.41 (−0.42, 086)

* *p* < 0.05. C_min_ = trough serum concentration; C_max_ = peak serum concentration; t_1/2_ = half-life; Δ = change in serum concentration from trough to peak.

## Data Availability

Upon request from authors.
